# Insight into the long-term impact of birth weight on intestinal development, microbial settlement, and the metabolism of weaned piglets

**DOI:** 10.1093/jas/skad395

**Published:** 2023-12-08

**Authors:** Paolo Trevisi, Clara Negrini, Federico Correa, Sara Virdis, Luca Laghi, Mele Marcello, Giuseppe Conte, Maurizio Mazzoni, Diana Luise

**Affiliations:** Department of Agricultural and Food Sciences, University of Bologna, Bologna, Italy; Department of Agricultural and Food Sciences, University of Bologna, Bologna, Italy; Department of Agricultural and Food Sciences, University of Bologna, Bologna, Italy; Department of Agricultural and Food Sciences, University of Bologna, Bologna, Italy; Department of Agricultural and Food Sciences, University of Bologna, Cesena, Italy; Department of Agriculture, Food and Environment, University of Pisa, Pisa, Italy; Department of Agriculture, Food and Environment, University of Pisa, Pisa, Italy; Department of Veterinary Medical Sciences, University of Bologna, Ozzano Emilia, Italy; Department of Agricultural and Food Sciences, University of Bologna, Bologna, Italy

**Keywords:** amino acids, gut health, immunity, metabolome, T-lymphocytes

## Abstract

Infant mortality of low birth body weight (**LBBW**) piglets can reach 10% and is mainly due to gut and immune system immaturity which can lead to a higher risk in the long term. This study aimed to assess the impact of birth body weight (**BBW**) on piglet metabolism, gut status, and microbial profile from weaning to 21 d postweaning. At birth, 32 piglets were selected for their BBW and inserted into the normal BBW (**NBBW**:1.38 ± 0.09 g) or the LBBW (0.92 ± 0.07 g) group. The piglets were weighed weekly from weaning (d0) to d21. At d9 and d21, 8 piglets/group were slaughtered to obtain the distal jejunum for morphology, immunohistochemistry, and gene expression analysis, colon content for microbiota and short-chain fatty acid (**SCFA**) analysis, and intestinal content for pH measurement. Blood was collected for metabolomic, haptoglobin (**Hp**), and reactive oxygen metabolite (**ROM**) analysis. The LBBW group had a lower body weight (**BW**) throughout the study (*P* < 0.01), a lower average daily gain from d9-d21 (*P* = 0.002), and lower feed intake (*P* = 0.02). The LBBW piglets had lower Hp at d9 (*P* = 0.03), higher ROMs at d21 (*P* = 0.06), and a net alteration of the amino acid (AA) metabolism at d9 and d21. A higher expression of *NFKB2* was observed in the LBBW piglets at d9 (*P* = 0.003) and d21 (*P* < 0.001). *MYD88* expression was enhanced in NBBW piglets at d9 (*P* < 0.001). The LBBW piglets had a lower villus height, absorptive mucosal surface (*P* = 0.01), and villus height:crypt depth ratio (*P* = 0.02), and a greater number of T-lymphocytes in both the epithelium and the crypts (*P* < 0.001) at d21. At d21, the LBBW piglets had higher lactic acid, acetate, butyrate, and valerate, and also higher SCFA in the colon (*P* < 0.05). The LBBW piglets had a higher Shannon index (*P* = 0.01) at d9 and a higher abundance of SCFA-fermenting bacteria. In conclusion, the present study confirmed that LBBW could impact the gut mucosal structure, immunity, and inflammatory and oxidative status, leading to an altered AA metabolism, and delaying the recovery from weaning.

## Teaser

Piglets birth’ body weights have a strong impact on the long-term development of animals.

Low birth body weight piglets showed a higher degree of postweaning intestinal immaturity.

## Introduction

Birth body weight (**BBW**) is an important indicator and prognostic factor for neonatal health and growth in both the short and the long term. Piglets born with a weight below 1.1 kg are referred to as low birth body weight (**LBBW**) and are characterized by reduced body weight (**BW**) throughout their entire life Li et al., 2018). This condition is also associated with reduced feed intake (**FI**), resulting in reduced nutrient supply (De Vos et al., 2014), and delayed development of intestinal morphology (Michiels et al., 2013). Studies have confirmed that piglets born with lower birth weight are also associated with reduced immune competence, higher T-cell count, increased incidence of diseases or lack of immunoglobulin in the serum (Bæk et al., 2020; Prims et al., 2016; Rodrigues et al., 2022). In addition, delayed digestive function has been observed despite the distress connected with the weaning process (Michiels et al., 2013). LBBW piglets are commonly reared in the pig farming system, and their frequency has been increasing in recent years due to the genetic selection of highly prolific sows. Furthermore, LBBW piglets have a higher probability of mortality compared to normal BBW (**NBBW**) piglets (Hawe et al., 2020; Riddersholm et al., 2021). Weaning weight may be a more fundamental determinant than nursery survivability; however, birth weight is part of the variability in weaning weight (Quiniou et al., 2002), therefore, the relationship between birth weight and weaning weight is implicated through its influence on weaning weight (Fix et al., 2010). Furthermore, Novais et al., (2020) found that LBBW piglets are more exposed to oxidative damage in the jejunum, confirming that piglets born with lower birth weight experience metabolic and oxidative stress, especially during the pre-weaning period Novais et al., (2020). In addition, intrauterine growth restriction (**IUGR**) and low BBW piglets both suffer from metabolic disturbances, although direct comparisons were not made. Specifically, studies in piglets have shown a disturbance in the amino acid (**AA**) metabolism with a decrease in AA concentration, especially in branched-chain amino acids, in both the placenta and the blood of the pig, (Regnault et al., 2002), as well as a disturbance in the fatty acid metabolism (Ying et al., 2017). This impairment in the AA and lipid metabolism can consequently lead to metabolic diseases throughout their later life (Salam et al., 2014).

Although there is a plethora of studies on LBBW piglets in literature, most have focused on the pre-weaning period. In this study, it was hypothesized that BBW may have a long-term effect on the intestinal maturation of piglets, and that piglets born underweight may have increased intestinal inflammation, reduced absorptive capacity, and dysbiotic microbiota.

To define appropriate therapies and interventions useful to limit the consequences of this condition, such as morbidity and mortality, an understanding of the impact of BBW on the development of the intestinal structure, immune system, and microbial settlement is crucial. Therefore, the aim of this study was to evaluate the impact of BBW on piglet performance, intestinal morphological and immunological architecture, microbial profile, and blood metabolism from weaning to 21 d postweaning.

## Material and Methods

The in vivo trial was approved by the Ethics Committee for Experiments on Animals of the University of Bologna, Italy, and by the Italian Ministry of Health (Authorization no. 287/2021-PR released in compliance with art. 31 of the D.lgs. 26/2014), and complied with the Animal Research Reporting of In Vivo Experiments (ARRIVE) guidelines (du Sert et al., 2020).

About 32 piglets (20 females and 12 males) were selected from 13 multiparous sows (5 ± 1.49 parity order) from a farm located in northern Italy. At birth, the piglets were ear tagged and individually weighed and classified as low birth body weight (**LBBW**: 0.92 ± 0.07 g; *n* = 16) and NBBW (1.38 ± 0.09 g, *N* = 16). No creep feed was supplied during the suckling phase. At weaning (d0), the 32 piglets were transported from the farrowing unit to the experimental facility of the University of Bologna.

At d0, the LBBW and NBBW piglets were weighed and divided into 8 pens per group (2 piglets/pen) according to their class of BBW and balanced for BW at d0 and litter of origin. Feed was provided ad libitum and the piglets had continuous access to water. The calculated and analyzed dietary contents are reported in Supplementary Table S1 (CARRA Mangimi S.P.A., 43058 Sorbolo (PR), Italy). Each pen was equipped with enrichment material (a metal chain and a natural cotton rope) and the room temperature and humidity were recorded daily. The temperature varied from 30 °C at d0 to 28 °C at d21 and was additionally heated by infrared lamps for the first 7 d.

### Data and sample collection

The piglets were individually weighed weekly from d0 to d21 postweaning (end of the study). FI for each replicate was recorded daily. Fecal consistency based on a 5-point scale (1 hard feces—5 watery feces (Luise et al., 2019)) was recorded daily for each piglet.

Blood samples were collected at d9 and d21, using venipuncture of the vena cava of the piglets, obtaining serum using a collection tube without anticoagulant and a K3 EDTA (Vacutest Kima Srl, Arzergrande, PD, Italy) collection tube to obtain plasma. Before the blood collection, the piglets were fasted for 12 h, therefore the feed was removed from the feeder.

At d9 (acute phase) and d21, one piglet/replicate (total of 16 piglets per time point) was slaughtered according to the procedure described by Correa et al. (2022).

Immediately after the sacrifice, the intestinal contents of the distal jejunum, cecum, and colon were collected and diluted on a 1:1 ratio with deionized water (McDonnell et al., 2010) to determine the pH using a pH meter (Vio, Giorgio Bormac S.r.l., Carpi, MO, Italy). In addition, an aliquot of colonic content was immediately placed into a sterile tube, snap-frozen in liquid nitrogen, and then stored at −80 °C for microbiota and short-chain fatty acid (**SCFA**) analysis. Two samples of distal jejunal mucosa were collected, the first one was immediately snap-frozen in liquid nitrogen and then stored at −80 °C for gene expression analysis, and the second was formalin fixed for immunohistochemistry and morphological analysis.

### Analysis of the blood parameters and the blood metabolome

Serum reactive oxygen metabolites (ROM**s**) and Haptoglobin (**Hp**) were quantified. Serum was obtained at room temperature after allowing the blood to clot for 2 h and then centrifuged at 3,000 × *g* for 10 min. The ROMs (mmol H_2_O_2_/L) were quantified colorimetrically using the d-ROMs test kit (Diacron International Sr1, Grosseto, Italy). Serum samples were analyzed following Brambilla et al. (2002) procedure. Absorbance was read at 520 nm. The concentration of Hp (mg/mL) was determined by sandwich enzyme immunoassay using a Tridelta Phase Hp Assay Kit (Tridelta Development Ltd, Maynooth, Co., Kildare, Ireland), following the manufacturer’s instructions.

The metabolite profile of the blood plasma was evaluated. Blood was processed immediately after sampling by centrifugation of the tubes at 3,000 × *g* for 10 min at 4 °C, and the samples were stored at −80°C until metabolomic analysis. The ^1^H-NMR analyses were performed as previously described by Brugaletta et al. (2022).

### Jejunal gene expression analysis

An RNA Purification Kit (Thermo Fisher Scientific, Waltham, MA, USA) was used to extract the piglet jejunal mucosal total RNA, the residual DNA was removed using a TURBO DNA-free DNA Removal Kit (Thermo Fisher Scientific) and, finally, complementary DNA was obtained using a High-Capacity RNA-to-cDNA Kit (Thermo Fisher Scientific) as previously described by Luise et al. (2023). As a housekeeping gene, the hydroxymethylbilane synthase (*HMBS*) gene was used; nuclear factor kappa B subunit 2 (*NFKB2*), inhibitors of apoptosis (*IAP*), claudin-4 (*CLAUD4*), glutathione peroxidase 2 (*GPX-2*), interleukin-8 (*IL-8*), and myeloid differentiation primary response 88 (*MYD88*) genes were analyzed. The gene expression assay used is listed in Supplementary Table S2. The cycle threshold (Ct) value of the *HMBS* gene of each sample was obtained using the ΔΔCt method.

### Morphological and immunohistochemical analysis of the jejunal mucosa

For each piglet (32 in total), paraffin-embedded samples of jejunum were fixed for 48 h in 10% buffered formalin. Sections (7 µm thick) were stained with hematoxylin–eosin for morphological evaluation. The width and height of 30 villi, and the depth and the width of 30 crypts of each sample were measured as previously reported by Mazzoni et al. (2010).

For immunohistochemistry, the avidin–biotin–peroxidase complex (ABC) method was performed according to the procedures previously described by Mazzoni et al. (2018). The following antibodies were used: polyclonal rabbit anti-serum anti-CD3 diluted 1:1,500 (C7930, Sigma-Aldrich), polyclonal goat anti-porcine immunoglobulin A (IgA) diluted 1:2,000 (NB724, Novus Biologicals) and monoclonal mouse anti-Claudin-4 diluted 1:300 (32-9400, clone 3E2C1, ThermoFisher-Invitrogen). The following secondary antibodies were used goat anti-rabbit IgG, goat anti-mouse IgG, and horse anti-goat IgG, each diluted 1:200 (Vector). A Nikon Eclipse Ni microscope was used for morphometric evaluation, and the images were captured using a Nikon DS-Fi2 digital camera and NIS Elements software BR 4.20.01 (Nikon Instruments Europe BV, Amsterdam, The Netherlands). For each piglet, the following assessments were carried out: number and distribution of intraepithelial lymphocytes (**IEL**), and number of IgA-immunoreactive (**IgA-IR**) cells in the *lamina propria*, while the Claudin-4 immunoreactivity was evaluated by scoring. In detail, the IEL were evaluated in 10 villi and 10 crypts and in three different compartments adapted from Vega-López et al. (2001): 1) between enterocytes above the epithelial basal lamina; 2) in the *lamina propria* of the villus-axis; and 3) in the *lamina propria* interspersed with the crypts. The IgA cell quantitative analysis was performed and adapted to the method previously described by Bianco et al. (2014). The IgA-IR cell count was performed in the mucosal *lamina propria* of the villus-axis and the *lamina propria* intermingled with the crypts. The blood/lymphatic vessel area was manually excluded, and the villi/crypts IgA-IR cells were expressed as the number of IgA-IR cells/4,000 μm^2^. The Claudin-4 immunoreactivity was evaluated in 10 villi per piglet as previously described by Correa et al. (2022).

### Microbial and SCFA analysis of the colon

For the analyses of microbial communities, FastDNA SPIN Kit for Soil (MP Biomedicals, Santa Ana, CA, USA) was used to extract bacterial DNA from colon content, following the manufacturer’s instructions. The concentration and purity of the isolated DNA were assessed using spectrophotometry on NanoDrop (Fisher Scientific, Schwerte, Germany), measuring absorbance ratios at 260/280 and 260/230. The V3–V4 region of the 16S rRNA gene, ~460 base pairs in length, was amplified. Universal primers Pro341F: 5ʹ-TCGTCGGCAGCGTCAGATGTGTATAAGAGACAGCCTACGGGNBGCASCAG-3ʹ and Pro805R: 5ʹGTCTCGTGGGCTCGGAGATGTGTATAAGAGACAGGACTACNVGGGTATCTAATCC-3ʹ (Takahashi et al., 2014) were used for this purpose, along with Platinum Taq DNA Polymerase High Fidelity (Termo Fisher Scientific, Italy). Sequencing was performed on the Illumina MiSeq platform with 300 × 2 base pair reads. Libraries were prepared following the standard protocol for MiSeq Reagent Kit V3 and then sequenced on the MiSeq platform (Illumina Inc., San Diego, CA, USA). For the subsequent bioinformatics analysis, the DADA2 pipeline was employed (Callahan et al., 2016), utilizing the Silva database (Quast et al., 2013) version 138.1 for taxonomic assignment. Raw sequences had their primers removed, and forward and reverse reads were trimmed at positions 290 and 250, based on the average quality score. All other DADA2 settings were kept at their default values.

The SCFAs, including acetate, propionate, iso-butyrate, butyrate, valerate, and isovalerate, as well as lactic acid in the colon content samples, were analyzed using high-performance liquid chromatography (HPLC). The following procedures were followed: 5 g of content was mixed with 25 mL of 0.1 N H_2_SO_4_ aqueous solution and homogenized for 2 min using an UltraTurrax (IKA-Werke GmbH & Co. KG, Staufen, Germany) (Sandri et al., 2017). The mixture was then centrifuged at 5,000 × *g* for 15 min at 4 °C to separate the liquid phase from the solid residuals. The liquid phase was subsequently microfiltered using a 0.45-μm Millex-HV filter (Merck-Millipore, Billerica, MA). The resulting sample was directly injected into the HPLC, which included an Aminex 85 HPX-87 H ion exclusion column (300 mm × 7.8 mm; 9-μm particle size; Bio-Rad, Milan, Italy) and was maintained at 40 °C. The detection wavelength was set at 220 nm. The analyses were performed using an isocratic elution (flow rate of 0.6 mL/min) with a 0.008 N H_2_SO_4_ solution as the mobile phase, and the injection loop volume was 20 μL. Individual SCFA and lactic acid were identified by comparing their retention times to a standard solution containing 4.50 mg/mL of lactic acid, 5.40 mg/mL of acetic acid, 5.76 mg/mL of propionic acid, 7.02 mg/mL of butyric acid and iso-butyric acid, 8.28 mg/mL of valeric acid and iso-valeric acid in 0.1 N H_2_SO_4_ (Sigma-Aldrich, Milano, Italy; catalog numbers 69775, 338826, 402907, B103500, 58360, 75054, 129542, respectively). Quantification was done using an external calibration curve based on the standards described above (Sandri et al., 2017).

### Statistical and bioinformatic analysis

Statistical analysis was performed in R v3.6 using the packages “car” 3.1-1 (Fox and Weisberg, 2019), “lm4” 1.1-31 (Bates et al., 2015) and “lsmeans” 2.30-0 (Lenth, 2021). The average daily gain (**ADG**), fecal score, pH of the intestinal contents, ROMs, Hp, metabolomic compounds, gene expression, SCFA, morphological and immunohistochemical values were analyzed using a linear mixed model in which the class of BBW was included as a fixed factor, and litter of origin was included as a random factor. For the FI and gain-to-feed (**G:F**) ratio, the replicate (pen) was used as an experimental unit and the data were fitted with a linear model including the class of BBW as a factor. Tukey’s honestly significant difference test was carried out at a 95% confidence level (*P* < 0.05). For the metabolomic data, the concentrations of the molecules were transformed into normality using log, or Box and Cox transformation (Box and Cox, 1964) prior to univariate analysis.

The statistical analysis of microbial data was performed with R software using the packages phyloseq 1.38.0 (McMurdie and Holmes, 2011), vegan 2.6 (Dixon, 2003), and microbiomeMarker 1.0.2 (Cao et al., 2022). Data were normalized using total sum scaling “TSS” transformation. For the alpha diversity, the Chao1, Shannon, and Simpson diversity indices were calculated, and the differences between the classes of BBW were tested using a linear model including sequencing depth of the BBW category as a fixed factor and litter of origin as a random factor. For the beta diversity, the Bray Curtis distance was calculated and plotted using a non-metric multidimensional scaling (**NMDS**) plot. The differences between the classes of BBW were tested using a non-parametric permutational multivariate analysis of variance (PERMANOVA) model (Adonis test) with 999 permutations. A differential abundance analysis was carried out using linear discriminant analysis effect size (**LEfSe**) implemented in the wrapper function included in the microbiomeMarker 1.0.2 package, aggregating the data at the genus level. A linear discriminant analysis (**LDA**) score of 3 and P.adj < 0.05 were used as a cutoff to detect the microbial markers of the two classes of BBW (Segata et al., 2011).

A significant effect was declared at *P* < 0.05, and *P* > 0.05 and  < 0.10 was considered a tendency.

## Results

### Health and performance

The piglets remained healthy throughout the study, and none was treated for severe health impairments. The fecal score of the piglets during the trial and the effect of the class of BBW are reported in Table 1. The weekly fecal score ranged from 2.00 to 2.15, showing no issues in the piglets caused by diarrhea; the BBW class did not affect the fecal consistency at any period.

**Table 1.** Effect of different birth body weight on fecal score of postweaning piglets

Fecal score	BBW class^1^		SEM	*P* value^2^
	LBBW	NBBW		
d0 to d7	2.13	2.12	0.04	0.81
d0 to d9	2.14	2.13	0.04	0.69
d0 to d14	2.10	2.13	0.03	0.55
d0 to d21	2.07	2.09	0.02	0.53
d9 to d14	2.04	2.01	0.02	0.32
d9 to d21	2.02	2.00	0.01	0.29

^1^The classes of birth body weight were divided into 2 groups: normal birth body weight (NBBW: 16 piglets, 1.38 ± 0.09 kg) and low birth body weight (LBBW: 16 piglets, 0.92 ± 0.07 kg).

^2^Statistical analysis were performed as follow: linear mixed model and ANOVA analysis in which the class of BBW was included as a fixed factor, and litter of origin was included as a random factor. Piglets were used as experimental unit.

The effect of BBW on growth performance parameters is reported in Table 2. The BBW significantly affected the BW at each time point (*P* < 0.001), and the NBBW piglets always had a higher BW compared to the LBBW piglets. The ADG for the periods d0 to d7, d0 to d9, d0 to d14, and d9 to d14 did not differ between the LBBW and the NBBW piglets; the ADG for the periods d0 to d21 and d9 to d21 was higher in the NBBW piglets as compared to the LBBW piglets (*P* = 0.048 and *P* = 0.002, respectively). The FI for the periods d0 to d7, d0 to d9, and d0 to d14 did not differ between the LBBW and the NBBW piglets; the FI for the periods d0 to d21 and d9 to d21 were higher in the NBBW piglets as compared with the LBBW piglets (*P* = 0.01 and *P* = 0.02, respectively), and the FI for the period d9 to d14 tended to be higher in the NBBW piglets as compared with the LBBW piglets (*P* = 0.09). The G:F ratio was never affected by the class of BBW.

**Table 2.** The effect of birth body weight on the growth performance of the piglets in the postweaning period

Item	BBW class^1^		SEM	*P* value
	LBBW	NBBW		
BW (g)^2^				
d0	6,359	7,880	211	<0.001
d7	6,686	8,112	240	<0.001
d9	6,837	8,320	232	<0.001
d14	8,162	10,027	536	<0.001
d21	10,972	13,252	634	<0.001
Average daily gain (g/day)^2^				
d0 to d7	49.8	33.8	13.6	0.40
d0 to d9	55.6	49.0	9.60	0.62
d0 to d14	132	163	27.5	0.31
d0 to d21	219	263	23.5	0.048
d9 to d14	258	367	64.6	0.13
d9 to d21	331	423	36.9	0.002
Feed intake (g)^3^				
d0 to d7	95.8	94.4	9.2	0.91
d0 to d9	111	119	8.2	0.47
d0 to d14	193	231	21.7	0.12
d0 to d21	317	378	27.5	0.01
d9 to d14	332	428	51.5	0.09
d9 to d21	468	570	46.6	0.02
Gain to feed ratio^3^				
d0 to d7	0.45	0.32	0.14	0.49
d0 to d9	0.45	0.39	0.08	0.61
d0 to d14	0.66	0.67	0.08	0.89
d0 to d21	0.68	0.69	0.02	0.50
d9 to d14	0.74	0.83	0.08	0.47
d9 to d21	0.70	0.74	0.02	0.17

^1^The classes of birth body weight were divided into 2 groups: normal birth body weight (NBBW: 16 piglets, 1.38 ± 0.09 kg) and low birth body weight (LBBW: 16 piglets, 0.92 ± 0.07 kg).

^2^Statistical analysis were performed as follow: linear mixed model and ANOVA analysis in which the class of BBW was included as a fixed factor, and litter of origin was included as a random factor. Piglets were used as experimental unit.

^3^Statistical analysis were performed as follows: the data were fitted using a linear model including the class of BBW as a factor followed by ANOVA analysis. The replicate (pen) was used as an experimental unit.

### Blood parameters and metabolome

The effect of BBW on the concentration of ROMs and haptoglobin is reported in Table 3. The BBW did not affect the ROM concentration at d9; however, it tended to influence the ROMs at d21 when the LBBW piglets had a higher ROM concentration as compared with the NBBW piglets (*P* = 0.06). The haptoglobin concentration was higher in the NBBW piglets as compared with the LBBW piglets at d9 (*P* = 0.03), while it did not differ at d21.

**Table 3.** The effect of birth body weight on the reactive oxygen metabolites and haptoglobin of piglets’ blood at 9- and 21-d postweaning

Item	BBW class^1^		SEM	*P* value^2^
	LBBW	NBBW		
ROMs^3^ (mmol H_2_O_2_/L)				
d9	28.7	29.4	1.9	0.82
d21	26.7	21.6	1.9	0.06
Haptoglobin (mg/mL)				
d9	1.79	2.26	0.15	0.03
d21	0.99	0.42	0.30	0.11

^1^The classes of birth body weight were divided into 2 groups: normal birth body weight (NBBW: 16 piglets, 1.38 ± 0.09 kg) and low birth body weight (LBBW: 16 piglets, 0.92 ± 0.07 kg).

^2^Statistical analysis were performed as follow: linear mixed model and ANOVA analysis in which the class of BBW was included as a fixed factor, and litter of origin was included as a random factor. Piglets were used as experimental unit.

^3^Reactive oxygen metabolites.

To study the changes in the plasma metabolome, the ^1^H-NMR spectra were recorded, and a total of 44 assigned molecules were quantified. Plasma metabolome results are presented in Supplementary Table S3 and Table 4. Table 4 reports the effect of BBW on the metabolome profile at d9 and d21. At d9, the LBBW piglets had a higher concentration of formate, beta-alanine, and alanine (*P* = 0.03, *P* = 0.02, *P* = 0.09, respectively) and a lower concentration of histidine, dimethyl sulfone, aspartate, and leucine compared to the NBBW piglets (*P* = 0.02, *P* = 0.04, *P* = 0.003, *P* = 0.001, respectively). Furthermore, tendencies for the BBW category were observed in tyrosine, choline, and valine (*P* = 0.06, *P* = 0.09, *P* = 0.07, respectively) which were lower in the LBBW piglets. At d21, differences were observed for lactate, 2-aminobutyrate, and leucine (*P* = 0.005, *P* = 0.07, *P* = 0.07, respectively), which were higher in the LBBW piglets, while trans-4-Hydroxy-L-proline and serine (*P* = 0.03, *P* = 0.005, respectively), were lower in the LBBW piglets as compared with the NBBW piglets. Furthermore, tendencies were observed for mannose, threonine, glycine, and beta-alanine (*P* = 0.08, *P* = 0.08, *P* = 0.09, *P* = 0.06, respectively) which were lower in the LBBW piglets as compared with the NBBW piglets.

**Table 4.** The effect of birth body weight on the plasma metabolome of piglets at d9 and d21 postweaning

Metabolite	d9				d21			
	BBW class^1^		SEM	*P* value^2^	BBW class^1^		SEM	*P* value^2^
	LBBW	NBBW			LBBW	NBBW		
2-Aminobutyrate	0.14	0.1	0.02	0.12	0.07	0.05	0.01	0.07
Alanine	4.29	3.64	0.33	0.09	2.77	2.67	0.05	0.92
Aspartate	0.47	0.59	0.06	0.003	0.53	0.56	0.01	0.91
Beta-alanine	10.03	5.74	2.15	0.02	9.54	10.93	0.69	0.06
Choline	0.05	0.07	0.01	0.09	0.07	0.05	0.01	0.17
Dimethyl sulfone	0.06	0.09	0.01	0.04	0.07	0.09	0.01	0.38
Formate	0.41	0.27	0.07	0.03	0.41	0.40	0.004	0.93
Glycine	3.87	4.48	0.31	0.19	3.76	4.37	0.31	0.09
Histidine	0.11	0.26	0.08	0.02	0.08	0.06	0.01	0.42
Lactate	15.21	14.15	0.53	0.50	22.84	14.66	4.09	0.005
Leucine	0.53	0.66	0.07	0.001	0.51	0.35	0.08	0.07
Mannose	0.42	0.45	0.02	0.12	0.40	0.45	0.03	0.08
Serine	0.68	0.71	0.02	0.69	0.69	0.89	0.1	0.005
Threonine	0.87	0.92	0.02	0.43	1.10	1.25	0.07	0.08
trans-4-hydroxy-L-proline	0.24	0.26	0.01	0.46	0.24	0.29	0.03	0.03
Tyrosine	0.21	0.25	0.02	0.06	0.18	0.19	0.01	0.49
Valine	0.99	1.1	0.06	0.07	0.83	0.68	0.07	0.44

^1^The classes of birth body weight were divided into 2 groups: normal birth body weight (NBBW: 16 piglets, 1.38 ± 0.09 kg) and low birth body weight (LBBW: 16 piglets, 0.92 ± 0.07 kg).

^2^Statistical analysis were performed as follow: linear mixed model and ANOVA analysis in which the class of BBW was included as a fixed factor, and litter of origin was included as a random factor. Piglets were used as experimental unit. Prior to analyses, the log, or Box and Cox transformation was performed to normalize the data distribution.

### Jejunal gene expression, morphology, and immunohistochemical measurements

The effect of BBW on the expression of *NFKB2*, *IAP*, *CLAUDIN-4*, *GPX-2, IL-8,* and *MYD88* is reported in Table 5. The class of BBW did not affect the gene expression at d9 and at d21, except for the *NFKB2* and *MYD88* expression. *NFKB2* was higher in the LBBW piglets as compared to the NBBW piglets at d9 and d21 (*P* = 0.003 and *P* < 0.001, respectively). Meanwhile, the expression of *MYD88* was increased in the NBBW piglets at d9 (*P* < 0.001).

**Table 5.** The effect of birth body weight on the jejunal gene expression of piglets at d9 and d21 postweaning

Item	BBW class^1^		SEM	*P* value^2^
	LBBW	NBBW		
*d9*				
*IL-8*^3^	0.04	0.02	0.07	0.77
*MYD88*^4^	-0.03	0.12	0.03	<0.001
*NFKB2*^5^	0.13	-0.04	0.06	0.003
*IAP*^6^	-0.22	-0.09	0.07	0.24
*CLAUD4*^7^	0.006	0.08	0.08	0.57
*GPX-2*^8^	0.09	0.03	0.08	0.56
d21				
*IL-8*^3^	-0.12	-0.12	0.07	0.99
*MYD88*^4^	0.07	0.04	0.02	0.32
*NFKB2*^5^	0.08	-0.01	0.04	<0.001
*IAP*^6^	0.22	0.14	0.11	0.64
*CLAUD4*^7^	0.19	0.12	0.06	0.35
*GPX-2*^8^	-0.009	-0.02	0.12	0.91

^1^The classes of birth body weight were divided into 2 groups: normal birth body weight (NBBW: 16 piglets, 1.38 ± 0.09 kg) and low birth body weight (LBBW: 16 piglets, 0.92 ± 0.07 kg).

^2^Statistical analysis were performed as follow: linear mixed model and ANOVA analysis in which the class of BBW was included as a fixed factor, and litter of origin was included as a random factor. Piglets were used as experimental unit. Prior to analyses, the log, or Box and Cox transformation was performed to normalize the data distribution.

^3^Interleukin-8.

^4^Myeloid differentiation primary response 88.

^5^Nuclear factor kappa B subunit 2.

^6^Inhibitors of apoptosis.

^7^Claudin-4.

^8^Glutathione peroxidase 2.

**Figure 1.**
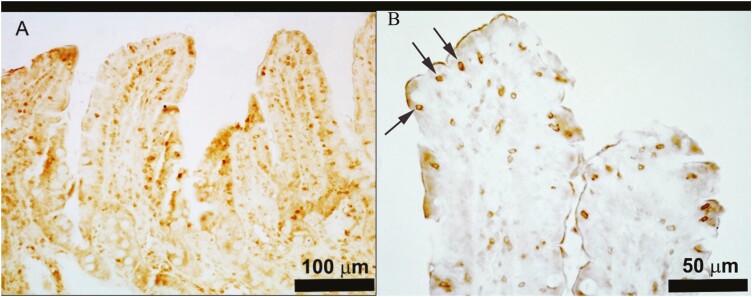
Representative images of porcine jejunal villi stained with anti-CD3 antibody. The immunoreactive T-lymphocytes were widely distributed both in the epithelial layer and in the lamina propria connective tissue (A and B). The high magnification in B shows the immunoreactive T-lymphocytes intermingled with enterocytes at the top of the villi (arrows).

**Figure 2.**
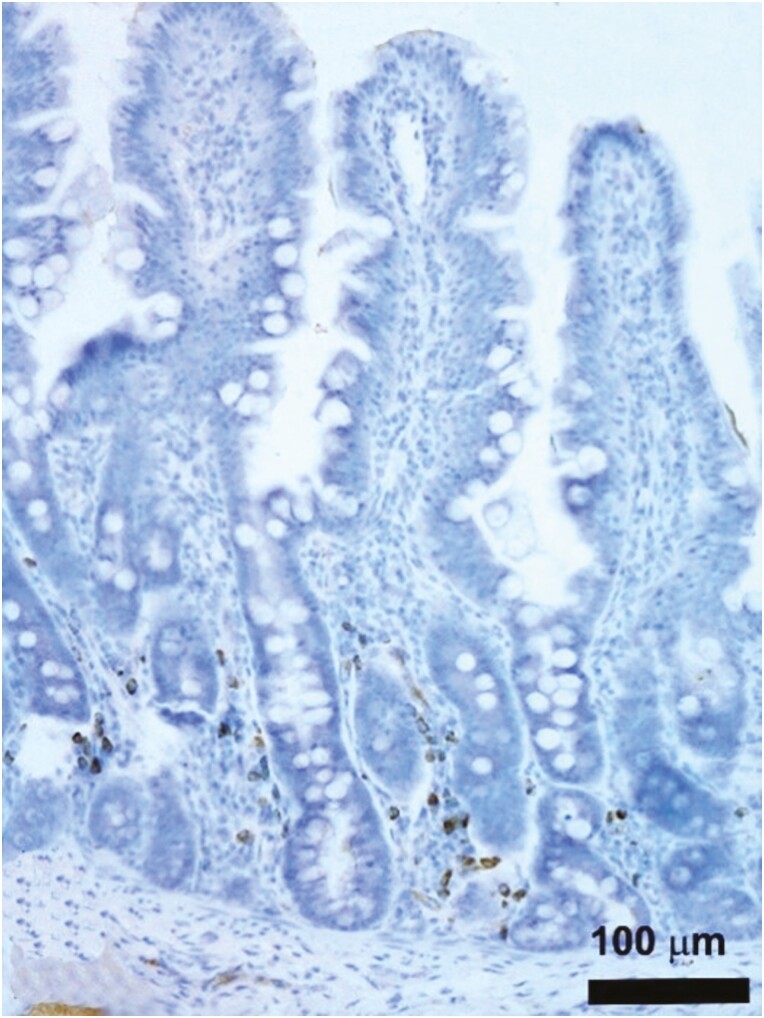
Representative image of porcine jejunal mucosa stained with anti-IgA antibody. The majority of the immunoreactive IgA producing cells were densely packed between the loose connective tissue surrounding the intestinal gland. The immunoreaction was counterstained with toluidine blue.

The effect of BBW on gut morphological and immune-histological parameters is reported in Table 6. At d9, the villus width (*P* = 0.01) and the IgA in the crypts (*P* = 0.007) were lower in the LBBW piglets as compared to the NBBW piglets. At d21, the villus width, crypt width, crypt depth, the number of IEL in the villi, the IgA in the crypts, and the Claudin-4 score were not affected by the class of BBW. At d21, the villus height (*P* = 0.01), absorptive mucosal surface (*P* = 0.01), and villus height to crypt depth (**VH:CD**) ratio (*P* = 0.02) were lower in the LBBW piglets as compared to the NBBW piglets. Furthermore, the number of IEL in the epithelium (*P* < 0.001) and crypts (*P* < 0.001) was higher (Figure 1A and B), and the IgA in the villi tended to be lower (*P* = 0.09) in the LBBW piglets as compared with the NBBW piglets (Figure 2).

**Table 6.** The effect of birth body weight on the jejunum morpho-immunohistological parameters of piglets at d9 and d21 postweaning

Item	BBW class^1^		SEM	*P* value^2^
	LBBW	NBBW		
d9				
Villus height, µm	177	194	12.6	0.33
Villus width, µm	80.9	94.5	3.7	0.01
Crypt width, µm	39.2	38.8	2.0	0.89
Crypt depth, µm	177	188	6.9	0.26
Absorptive mucosal surface	4.37	4.86	0.23	0.11
VH:CD ratio^3^	1.00	1.04	0.07	0.69
T-lymphocytes epithelium^4^	9.90	7.73	1.00	0.13
T-lymphocytes villus^4^	10.9	11.20	1.40	0.87
T-lymphocytes crypt^4^	8.94	7.17	0.90	0.15
IgA villus^4^	1.26	1.30	0.10	0.83
IgA crypt^4^	2.81	4.02	0.30	0.007
Claudin-4 score	2.00	2.00	0.20	1.00
d21				
Villus height, µm	204	264	16.1	0.01
Villus width, µm	106	111	4.30	0.36
Crypt width, µm	37.8	38.2	0.94	0.77
Crypt depth, µm	206	203	8.40	0.76
Absorptive mucosal surface	4.69	5.82	0.31	0.01
VH:CD ratio	0.99	1.31	0.10	0.02
T-lymphocytes epithelium^4^	10.37	6.74	0.40	<0.001
T-lymphocytes villus^4^	9.94	9.35	1.10	0.69
T-lymphocytes crypt^4^	6.76	4.59	0.50	<0.001
IgA villus^4^	1.17	1.76	0.30	0.09
IgA crypt^4^	4.47	4.51	0.30	0.93
Claudin-4 score	1.43	1.75	0.20	0.33

^1^The classes of birth body weight were defined into 2 groups: normal birth body weight (NBBW: 16 piglets, 1.38 ± 0.09 kg) and low birth body weight (LBBW: 16 piglets, 0.92 ± 0.07 kg).

^2^Statistical analysis were performed as follow: linear mixed model and ANOVA analysis in which the class of BBW was included as a fixed factor, and litter of origin was included as a random factor. Piglets were used as experimental unit.

^3^VH:CD ratio: villus height to crypt depth ratio.

^4^Number expressed on a 4,000 μm^2^ area.

### Intestinal pH, colon SCFAs, and microbial profile

The effect of BBW on the intestinal pH and the concentration of SCFAs in the colon is reported in Tables 7 and 8. The class of BBW did not affect the pH of the jejunum, cecum, and colon at d9, and the pH of the jejunum and cecum at d21. The LBBW piglets had a higher pH in the colon as compared to the NBBW piglets at d21 (*P* = 0.001).

**Table 7.** The effect of birth body weight on the pH of the intestinal contents of piglets at d9 and d21 postweaning

pH	BBW class^1^		SEM	*P* value^2^
	LBBW	NBBW		
d9				
Jejunum	7.47	7.44	0.09	0.63
Cecum	6.46	6.36	0.16	0.62
Colon	6.66	6.59	0.14	0.71
d21				
Jejunum	7.56	7.58	0.07	0.84
Caecum	6.80	6.99	0.09	0.14
Colon	6.90	6.68	0.07	0.001

^1^The classes of birth body weight were divided into 2 groups: normal birth body weight (NBBW: 16 piglets, 1.38 ± 0.09 kg) and low birth body weight (LBBW: 16 piglets, 0.92 ± 0.07 kg).

^2^Statistical analysis were performed as follow: linear mixed model and ANOVA analysis in which the class of BBW was included as a fixed factor, and litter of origin was included as a random factor. Piglets were used as experimental unit.

**Table 8.** The effect of birth body weight on the short-chain fatty acid (SCFA) concentration in the colons of piglets at d9 and d21 postweaning

Fatty acid, (mg/g)	BBW class^1^		SEM	*P* value^2^
	LBBW	NBBW		
d9				
Lactic acid	0.27	0.68	0.20	0.15
Acetate	8.93	7.41	1.30	0.32
Propionate	6.36	5.18	0.99	0.36
Butyrate	7.16	8.38	1.70	0.59
Iso-butyrate	2.24	4.17	0.88	0.11
Valerate	2.19	2.06	0.11	0.39
Isovalerate	3.33	5.95	1.10	0.09
Total	29.9	33.0	4.07	0.53
d21				
Lactic acid	2.09	0.15	0.70	0.007
Acetate	8.97	4.15	1.40	0.01
Propionate	4.75	3.98	0.60	0.35
Butyrate	12.18	4.41	3.10	0.01
Iso-butyrate	2.75	1.91	0.40	0.09
Valerate	2.15	1.85	0.07	0.003
Isovalerate	2.48	2.19	0.10	0.09
Total	33.8	17.6	3.80	<0.001

^1^The classes of birth body weight were divided into 2 groups: normal birth body weight (NBBW: 16 piglets, 1.38 ± 0.09 kg) and low birth body weight (LBBW: 16 piglets, 0.92 ± 0.07 kg).

^2^Statistical analysis were performed as follow: linear mixed model and ANOVA analysis in which the class of BBW was included as a fixed factor, and litter of origin was included as a random factor. Piglets were used as experimental unit.

At d9, the colon concentration of the total SCFA, and lactic, acetic, propionic, butyric, valeric, and iso-butyric acids were not affected by BBW; the iso-valeric concentration tended to be affected by the class of BBW, and the LBBW piglets tended to have a lower concentration of iso-valeric acids as compared to the NBBW piglets (*P* = 0.09). At d21, except for propionic acid, all the other SCFA and the total amount of SCFA were affected or tended to be affected (iso-butyrate and isovalerate) by the class of BBW; the LBBW piglets had a higher colon concentration of lactic acid (*P* = 0.007), acetic acid (*P* = 0.01), butyric acid (*P* = 0.01) and valeric acid (*P* = 0.003), and tended to have a higher concentration of iso-butyric and iso-valeric acids (*P* = 0.09) compared to the NBBW piglets.

Regarding the microbial profile, next-generation sequencing allowed obtaining a total of 1,447,928 raw reads which were attributed to a total of 6,803 amplicon sequence variants (**ASVs**). The relative rarefaction curve (Supplementary Figure S1) showed a tendency to plateau for all samples, suggesting that the sequencing depth was sufficient to describe the variability within the microbial communities analyzed. The ASVs allowed detecting of 21 phyla (Supplementary Figure S2A), 189 families (Supplementary Figure S2B), and 225 genera (Supplementary Figure S2C). The most abundant phyla belonged to Firmicutes 55 ± 0.11%, Bacteroidota 32 ± 0.09%, Spirochaetota 7 ± 0.06%, and Proteobacteria 2 ± 0.02%; the most abundant families were *Prevotellaceae* 19 ± 0.08%, *Lachnospiraceae* 17 ± 0.05%, *Spirochaetaceae* 7 ± 0.06%, and *Oscillospiraceae* 7 ± 0.03% and the most abundant genera were *Prevotella* 11 ± 0.07%, *Treponema* 7 ± 0.06%, and *Lactobacillus* 5 ± 0.06%.

**Figure 3.**
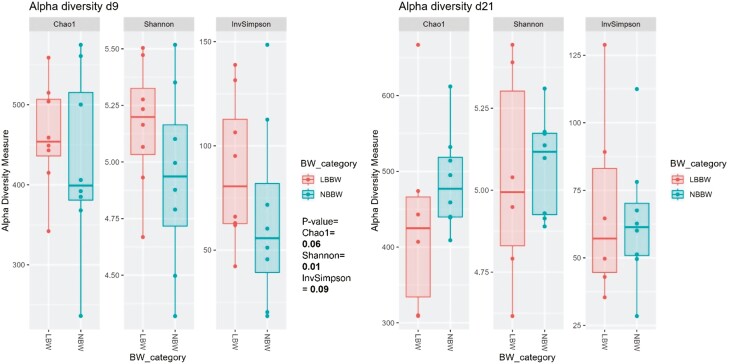
The effect of birth body weight on the Chao1, Shannon and InvSimpson index values of the piglet cecum samples obtained at days nine and twenty-one postweaning. BBW class: the classes of birth body weight were divided into 2 groups: normal birth body weight (NBBW: 16 piglets, 1.38 ± 0.09 kg) and low birth body weight (LBBW: 16 piglets, 0.92 ± 0.07 kg). Statistical analyses were performed as follow: linear mixed model and ANOVA analysis in which the sequencing depth of the class of BBW was included as a fixed factor, and litter of origin was included as a random factor. Piglets were used as experimental unit.

**Figure 4.**
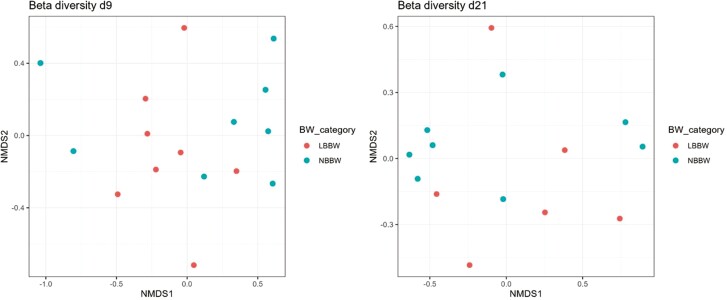
Effect of birth body weight on microbial beta diversity of weaned piglets at two timepoints. BBW class: the classes of birth body weight were divided into 2 groups: normal birth body weight (NBBW: 16 piglets, 1.38 ± 0.09 kg) and low birth body weight (LBBW: 16 piglets, 0.92 ± 0.07 kg). Statistical analyses were performed as follow: the Bray Curtis distance was calculated and plotted using a non-metric multidimensional scaling (NMDS) plot. The differences between the classes of BBW were tested using a non-parametric PERMANOVA model (Adonis test) with 999 permutations. Piglets were used as experimental unit.

**Figure 5.**
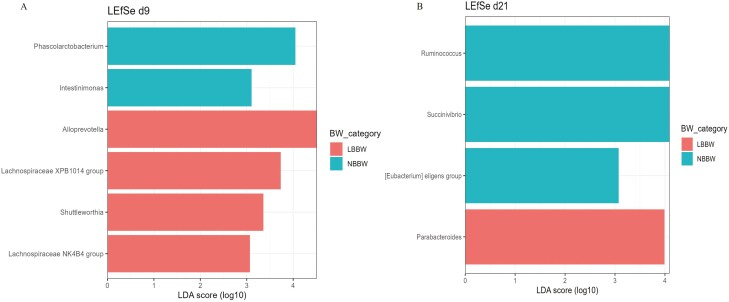
Linear discriminant analysis effect size (LEfSe) plots of the biomarker taxa identified in the piglet cecum samples, for each birth body weight category at days 9 (A) and 21 (B) postweaning. BBW class: the classes of birth body weight were divided into 2 groups: normal birth body weight (NBBW: 16 piglets, 1.38 ± 0.09 kg) and low birth body weight (LBBW: 16 piglets, 0.92 ± 0.07 kg). Statistical analyses were performed as follow: a linear discriminant analysis Effect Size (LEfSe) implemented in the wrapper function included in the microbiomeMarker 1.0.2 package was used, aggregating the data at the genus level. A linear discriminant analysis (LDA) score of 3 and *P*.adj < 0.05 were used as a cutoff to detect the microbial markers of the two classes of BBW. Piglets were used as experimental unit.

At d9, of the alpha diversity indices, the LBBW piglets had a higher Shannon index (*P* = 0.01) and tended to have higher Chao and Invsimpson indices (*P* = 0.06; *P* = 0.09, respectively) as compared to the NBBW piglets (Figure 3). No differences in the alpha diversity indices at d21 and no differences in the beta diversity index at d9 and d21 (Figure 4) were observed between the NBBW and the LBBW piglets. To identify specific bacterial markers that were differentially expressed between the LBBW and the NBBW piglets, a LEfSe was carried out, and the results are reported in Figure 5A and B for d9 and d21, respectively. At d9, the LBBW piglets were characterized by a higher abundance of *Alloprevotella* (LDA score = 4.45, *P* adj. = 0.02), Lachnospiraceae XPB1014 group (LDA score = 3.79, *P* adj. = 0.006), *Shuttleworthia* (LDA score = 3.42, *P* adj. = 0.001), and Lachnospiraceae NK4B4 group (LDA score = 3.01, *P* adj. = 0.04), while the NBBW piglets were characterized by a higher abundance of *Phascolarctobacterium* (LDA score = 3.98, *P* adj. = 0.05) and *Intestinimonas* (LDA score = 3.05, *P* adj. = 0.004). At d21, the LBBW piglets were characterized by a higher abundance of Parabacteroides (LDA score = 4.14, *P* adj. = 0.04), while the NBBW piglets were characterized by a higher abundance of *Ruminococcus* (LDA score = 4.11, *P* adj. = 0.003), *Succinivibrio* (LDA score = 4.08, *P* adj. = 0.04), and *Eubacterium* eligens (LDA score = 3.14, *P* adj. = 0.01).

## Discussion

The aim of the present study was to evaluate the impact of BBW on piglets’ metabolism and physiology in terms of gut architecture, integrity, and immune development, as well as the microbial profile and blood metabolism from weaning to 21 d postweaning.

The data showed that the BBW had a strong effect on the weight of piglets later in life as confirmed by previous studies in pigs (Fix et al., 2010). The FI and the ADG were lower in the LBBW pigs, starting from the ninth and fourteen days postweaning, respectively, at the end of the phase which is generally recognized as the most acute inflammatory phase for piglets (Pluske, 2016). In fact, at weaning, multifactorial stressors, including social stress, dietary transition, and a drop in passive immunity, as well as an increase in microbiological challenges (Gresse et al., 2017), can lead to transient anorexia and a perturbation of gut health and impairment the equilibrium of the gut microbial ecosystem, which is mainly visible in the first ten days postweaning. Transient anorexia, or fasting, impacts intestinal physiology as observed by Priori et al. (2015). Acute phase proteins are considered good inflammatory markers; among them, Hp is widely used as a marker of inflammation (Correa et al., 2023). However, it is also used to detect both clinical and subclinical diseases in piglets (Sauerwein et al., 2005) and it is also involved in the oxidative status. In addition, the same authors observed that Hp is positively correlated with the ROM concentration and inversely correlated with BW in postweaning piglets (Sauerwein et al., 2005). In fact, Hp is connected to the prevention of the production of hydroxyl radicals and lipid peroxides and is hemoglobin-driven. A possible explanation for the higher Hp in the blood of NBBW piglets could be linked to higher antioxidant activity via the prevention of lipid peroxides; furthermore, a greater percentage of adipose tissue and fatty acid circulation could have been present in this category of pigs. Independently from the blood markers for the inflammation and oxidative stress, the gene expression of the jejunal mucosa showed an impact of BBW on the oxidative balance. To this point, the higher jejunal expression of *NFKB2* observed, both at d9 and d21, indicated the activation of oxidative stress-related pathways, leading to an increase in ROMs and nitrogen species, which can then lead to tissue damage and mitochondrial impairment (Poggi and Dani, 2018). The increase in the *NFKB2* jejunal expression in the LBBW piglets was in line with the trend of the higher ROM concentration found in the blood at d21 and was consistent with the studies performed on very low birth body weight (**VLBW**), preterm human infants, and human infants with necrotizing enterocolitis (NEC), although no direct comparison was made (Ng, 2014; Tirone et al., 2019). However, in the present study, no differences were observed in the *GPX2* expression, which is considered an additional marker of intestinal oxidative stress. Furthermore, the greater expression of *NFKB2* could also be linked to increased intestinal inflammation, as *NFKB2* is upregulated by pro-inflammatory cytokines (Liu et al., 2017). Moreover, it is conceivable that the alteration in *MYD88* expression in LBBW piglets could influence their immune response, thereby affecting their overall health. Indeed, Lessard et al. (2018) observed a downregulation of *MYD88* expression as well as other genes related to innate immune activation in piglets with a low weight gain. These results may indeed, confirm the lower maturity of the immune system in piglets born with a low BW.

Consistent with this, a higher number of intraepithelial T-cells in the epithelium and crypts of the LBBW piglets was observed at d21, suggesting a higher level of inflammation in this group of piglets. Intraepithelial T-cells are essential elements of the vertebrate immune systems, providing a highly targeted and long-lasting immunological response when the organism is threatened (Von Bochmer, 1997). Moreover, they are strictly regulated and have a regulatory functions to minimize inflammation, namely an essential function to ensure epithelial integrity and immunological stability (Montufar-Solis et al., 2007; Olivares-Villagómez and Van Kaer, 2018). In the present study, the LBBW piglets showed a higher number of T-cells at d21 postweaning when the T-cells were supposed to be more mature. An increase in the T-cells in the epithelium and villi at d9 in the LBBW piglets, and a general recovery, alongside a minor effect in this BBW category at d21 were expected. Instead, the increase observed in the T-cells in both the epithelium and the crypts at d21 still indicated greater activation of the immune system in the LBBW piglets than in the NBBW piglets, which may be associated with a slower recovery of the LBBW piglets from the acute inflammatory phase as a result of weaning.

Since intestinal T-cells are involved in maintaining homeostasis and regulating enterocyte physiology, morphometric analyses were performed in the present study. The results obtained showed that the LBBW piglets had a lower villus height, a lower VH:CD ratio, and a less absorptive mucosal surface at d21 which was consistent with the results of the T-cell count. Indeed, previous studies in piglets have reported that low BW is often associated with a lack of intestinal absorption (D’Inca et al., 2011; Wiyaporn et al., 2013).

The reduction in the exchange surface area and the absorptive mucosa in the LBBW piglets was critical because it resulted in a reduction in the intestinal uptake of dietary nutrients and available antigenic compounds. In agreement with He et al. (2011), in the present study, the metabolomic analysis showed a perturbation of the AA metabolism at both d9 and d21 postweaning. Already at d9, the LBBW piglets had a lower blood concentration of aspartate, histidine, leucine, valine, and tyrosine, and a higher concentration of beta-alanine. Amino acids are essential precursors of proteins; therefore, a perturbation of AA availability may have impaired body growth and development in the LBBW piglets; however, AA are also involved in a wide number of metabolic pathways and can act as signaling molecules that can modulate the gut health (Chalvon-Demersay et al., 2021). In the present study, the pigs were fasted 12 h before blood sampling, so the AA level could be considered a baseline. Specifically, beta-alanine has been implicated in the regulation of intestinal health in piglets, which is associated with increased villus height and the mitotic activity of intestinal cells (Chen et al., 2023). In addition, beta-alanine can also activate the *NFKB2* signaling pathway (Lan et al., 2020), which is in line with the results of the present study regarding this gene’s expression. Beta-alanine is closely connected to the availability of histidine since they are both involved in the synthesis and breakdown of carnosine in muscles; therefore, they are both involved in muscle and fat metabolism (Brosnan and Brosnan, 2020). In the present study, the higher beta-alanine and the lower histidine may have indicated a deficiency in carnosine production in the LBBW piglets’ muscles. In addition, histidine, leucine, and valine are important AA for piglets, and their limitation in the blood can both reduce the ADG and impair the gut health of the animals (Wessels et al., 2016). In particular, leucine is involved in muscle and energy metabolism and can regulate the absorption and utilization of valine and isoleucine (Shimomura and Harris, 2006; Mayneris-Perxachs and Swann, 2018). Interestingly, in a previous study based on a dose-response piglets’ feeding trial, it was observed that low BW postweaning piglets required more leucine to achieve the same performance as normal BW piglets (Bertocchi et al., 2019). Aspartate is another AA involved in gut health, in particular, in the increasing of villus height and of the VH:CD ratio in piglets (Luise et al., 2023a); therefore, a reduction in its circulation in LBBW piglets could be linked to the morphological results observed in the present study. In line with these results, the increase in formate concentration observed in the current trial at d9 could be linked to the alteration of the AA metabolism. Moreover, formate is a precursor of nucleotide synthesis as well as a growth-limiting factor (Tibbetts and Appling, 2010; Ducker and Rabinowitz, 2017). The imbalance in formate concentration could also be involved in an increase in the glycolysis and intracellular production of lactate under stressful conditions as observed in rats (Heynen et al., 2022). However, in the present study, a higher blood concentration of lactate was observed only at d21 in the LBBW piglets, indicating a mid-term effect of BBW on pig physiology. Lactate is also considered to be an important biomarker of the energy metabolism, as an increase in its circulation is associated with increased glycolysis and energy demand, especially under stressful and inflammatory conditions (Alinaghi et al., 2019). Therefore, the higher lactate concentration in the blood of the LBBW piglets could be related to the lower energy available owing to the lower FI and limited intestinal nutrient uptake capacity as shown by the previous results. In addition to the impairment in the energy metabolism, the LBBW piglets continued to have an alteration of the AA metabolism at d21, mainly characterized by a decrease in proline, serine, glycine, and threonine. Glycine, serine, and threonine metabolism are involved in the same pathway. Serine and threonine can be used to produce glycine; furthermore, they are involved in gut health; as an increase in threonine availability and uptake may favor villus height and VH:CD ratio of the piglets’ small intestine, and can regulate pro-inflammatory and anti-inflammatory interleukins via the mTOR pathway (Ren et al., 2014). In the present study, the lower concentration of threonine observed in the blood of the LBBW piglets was in line with the lower villus height, absorptive mucosal surface, and greater number of T-cells in the jejunum of the LBBW piglets. Furthermore, glycine, proline, and threonine are currently considered to be conditionally essential AA for piglets. They play an essential role in protein synthesis and, hence, in growth (Dervishi et al., 2021; Wellington et al., 2023); therefore, their lower concentration in the LBBW piglets’ blood could be linked to the reduced growth performance observed in the present study.

Taken together, the lower concentration of the AA observed at both d9 and d21, and the increase in lactate at d21 confirmed an alteration of the AA and the energetic metabolism of the LBBW piglets, suggesting that low BW at birth can have long-term detrimental effects (Gonzalez-Bulnes et al., 2016).

In addition, since the microbiome and its fermentative activity are strictly connected with the metabolism, gut functionality, and the immune and oxidative status of the host, the microbiota and the SCFA were analyzed in the colon of the piglets. Previous studies on LBBW and IUGR piglets have suggested that the alteration of the intestinal microbiome is associated with an alteration of the piglet metabolism and metabolome in early life (Li et al., 2018; Huang et al., 2020). In agreement with these studies, the results of the present study showed that a low BBW can impact the microbiota in later life. The alpha diversity indices suggested that the LBBW piglets had greater species diversity compared to the NBBW piglets at d9 but not at d21 postweaning. These results were in agreement with the observations reported by Li et al. (2018) in whose study the alpha diversity of LBBW piglets was higher during early life (suckling period), and with the study of Huang et al. (2020) in which no differences in the alpha diversity indices in the colon microbial profile of LBBW and NBBW piglets at 70 d of age were observed. This effect of time could be associated with the capacity of the intestinal microbial community to adapt to the postweaning diets. The most evident effect of BBW was observed early after weaning which is, as mentioned previously, the most critical period for gut health. Furthermore, the present finding demonstrated that BBW affected specific bacterial taxa at all time points. It was observed that the relative abundances of the *Lachnospiraceae* XPB1014 group, the *Lachnospiraceae* NK4B4 group, *Shuttleworthia,* and *Alloprevotella* were higher in the LBBW piglets. It is interesting to note that, in the earlier study of Huang et al. (2020), both the *Lachnospiraceae* XPB1014 group and the *Lachnospiraceae* NK4B4 group were found to be higher in the LBBW piglets. An increase in *Lachnospiraceae* XPB1014 was also observed in the colons of the piglets fed with a reduction in dietary crude protein (Zhao et al., 2020), indicating that a reduction in AA availability, as observed in the present study for the LBBW piglets, could affect its abundance. The higher concentrations of all the SCFA in the colon of the LBBW piglets could have been due to the reduced capacity of these animals to digest and absorb the nutrients which were more available substrate for commensal bacteria in the colon. In agreement with that, Thymann et al. (2009) and Di Lorenzo et al. (1995) hypothesized that, since preterm and NEC piglets are characterized by a lower capacity to digest carbohydrates, an increase in readily available sugars for microbial fermentation occurs. As a result, enhanced production of SCFA from carbohydrate fermentation is observed, leading to a bacterial overgrowth that can damage the intestinal epithelium. In agreement with these previous studies, the present results confirmed that, in the long term, also LBBW piglets can suffer from deficiencies similar to preterm and NEC piglets.

The present study confirmed that LBBW can have an impact on the gut mucosal structure, immunity, and inflammatory status as well as the oxidative status of the piglets in the long term. In addition, the observed higher degree of immaturity of the intestinal mucosa could have compromised the microbial colonization and the gut capacity to absorb the nutrients and SCFA, resulting in an alteration of the piglets’ metabolism, mainly visible by a reduction of the blood AA concentration. In turn, the alteration in the gut health and metabolism leads to the inability to recover weight compared to the piglets born with a normal weight. Overall, these results confirm the key importance of BBW for the programmed development of young animals and highlight the need to define specific dietary strategies to facilitate the recovery of the LBBW to increase their natural resistance to stressors and infections.

## Supplementary Material

skad395_suppl_Supplementary_Material

## Data Availability

Raw sequence data are available at the sequence reads archive (SRA) of NCBI https://www.ncbi.nlm.nih.gov/, PRJNA977203. The other datasets analyzed in this study are available from the corresponding author under reasonable request.

## Abbreviations

AA: amino acid
ABC: avidin–biotin–peroxidase complex
ADG: average daily gain
ASVs: amplicon sequence variants
BBW: birth body weight
BW: body weight
CLAUD4: claudin-4
FI: feed intake
G:F: gain to feed
GPX-2: glutathione peroxidase 2
HMBS: hydroxymethylbilane synthase
Hp: haptoglobin
HPLC: high-performance liquid chromatography
IAP: inhibitors of apoptosis
IEL: intraepithelial lymphocytes
Ig: immunoglobulin
IgA-IR: IgA immunoreactive
IL-8: interleukin-8
IUGR: intrauterine growth restriction
LBBW: low birth body weight
LEfSe: linear discriminant analysis effect size
MYD88: myeloid differentiation primary response 88
NBBW: normal birth body weight
NEC: necrotizing enterocolitis
NFKB2: nuclear factor kappa B subunit 2
NMDS: non-metric multidimensional scaling
ROM: reactive oxygen metabolite
SCFA: short-chain fatty acid
VH:CD: villus height to crypt depth ratio
