# Supplementary material for: Insight into the long-term impact of birth weight on intestinal development, microbial settlement, and the metabolism of weaned piglets
Source: J Anim Sci. 2023 Dec 8;101:skad395. doi: 10.1093/jas/skad395 (PMC10963063; doi:10.1093/jas/skad395)
Supplement: skad395_suppl_Supplementary_Material [file skad395_suppl_supplementary_material.docx]

**Supplementary Table S1.** Composition of pre-starter diet.

| ***Ingredients*** | | ***Inclusion*** |
| --- | --- | --- |
| Barley | % | 24.46 |
| Soybean meal 48% | % | 16.2 |
| Corn / barley flakes | % | 15 |
| Soft wheat | % | 10 |
| Corn | % | 10 |
| Milk whey dried | % | 6.5 |
| Wheat bran | % | 4.8 |
| Soybean oil | % | 2.5 |
| Spray dried porcine plasma | % | 2.5 |
| Potato protein concentrate | % | 1 |
| Soybean protein concentrate | % | 1 |
| Coconut oil | % | 0.8 |
| Dicalcium phosphate | % | 0.75 |
| Benzoic acid | % | 0.5 |
| L-lysine hcl | % | 0.42 |
| Salt | % | 0.4 |
| Citric acid | % | 0.3 |
| Calcium formate | % | 0.3 |
| Dl-methionine | % | 0.157 |
| Vitamin premix^1^ | % | 0.155 |
| L-threonine | % | 0.15 |
| Aroma | % | 0.1 |
| MINERAL PREMIX (no zinc)^2^ | % | 0.0801 |
| L-tryptophan | % | 0.07 |
| Val-ile-leu-his premix | % | 0.05 |
| Phytase | % | 0.0075 |
| **Calculated composition** | | |
| Dry matter | % | 89.97 |
| CP | % | 19 |
| FAT | % | 5.48 |
| CF | % | 3.74 |
| ASH | % | 4.5 |
| NDF | % | 14.1 |
| ADF | % | 4.6 |
| ADL | % | 0.77 |
| STARCH | % | 35.57 |
| SUGARS | % | 7.99 |
| LYS | % | 1.35 |
| MET | % | 0.43 |
| MET+CIST | % | 0.79 |
| TRP | % | 0.3 |
| THR | % | 0.9 |
| LEU | % | 1.5 |
| ISOL | % | 0.78 |
| VAL | % | 0.96 |
| HIST | % | 0.47 |
| ARG | % | 1.1 |
| Ca | % | 0.61 |
| P | % | 0.72 |
| Avail. P | % | 0.47 |
| Na | % | 0.25 |
| K | % | 0.82 |
| Cl | % | 0.51 |
| Mg | % | 0.12 |
| S | % | 0.15 |
| Fe | mg/kg | 160.7 |
| Cu | mg/kg | 106 |
| Zn | mg/kg | 149.9 |
| Mg | mg/kg | 84.38 |
| Se | mg/kg | 0.45 |
| J | mg/kg | 0.84 |
| BIOTIN | mg/kg | 0.15 |
| COLINE | mg/kg | 1163 |
| FOLIC ACID | mg/kg | 1.09 |
| VIT. PP | mg/kg | 54.89 |
| PANTHOT.AC. | mg/kg | 14.49 |
| VIT. A | UI/kg | 16000 |
| VIT. E | mg/kg | 105 |
| VIT. B1 | mg/kg | 4.76 |
| VIT. B2 | mg/kg | 5.59 |
| VIT. B6 | mg/kg | 6.2 |
| VIT. B12 | mg/kg | 0.02 |
| VIT. D3 | UI/kg | 1600 |
| LINOLEIC ACID | % | 2.44 |
| DIG.EN. | kcal/kg | 3435 |
| MET.EN. | kcal/kg | 3283 |
| NET EN. | kcal/kg | 2459 |
| LYS.SID | % | 1.25 |
| MET.SID | % | 0.4 |
| THR.SID | % | 0.27 |
| TRE.SID | % | 0.81 |
| M+C SID | % | 0.75 |
| VAL SID | % | 0.85 |
| ISOL.SID | % | 0.71 |
| LEU. SID | % | 1.36 |
| HIS. SID | % | 0.45 |
| ARG.SID | % | 1.01 |
| VIT. K 3 | mg/kg | 1.25 |

**Supplementary Table S2.** Information regarding genes, Assay ID and TaqMan assay catalogue for Real-Time PCR analysis.

| **Target gene** | **Complete name** | **Assay Id** | **TaqMan®** |
| --- | --- | --- | --- |
|  |  |  | **Gene Expression** |
|  |  |  | **Assay Catalogue n.** |
| *GPX-2* | Glutathione Peroxidase 2 | Ss03387478_u1 | 4351372 |
| *NFKB2* | Nuclear Factor Kappa B Subunit 2 | Ss06883741_g1 | 4331182 |
| *CLAUD4* | Claudin-4 | Ss03375006_u1 | 4351372 |
| *IAP* | Inhibitor of apoptosis | Ss06886550_g1 | 4351372 |
| *IL8* | Interleukin-8 | Ss03392435_m1 | 4331182 |
| *MYD88* | Myeloid differentiation primary response 88 | Ss03389125_m1 | 4331182 |
| *HMBS* | Hydroxymethylbilane Synthase | Ss03388782_g1 | 4351372 |

**Supplementary Table S3.** The effect of birth body weight on the plasma metabolome of piglets at days nine and twenty-one post weaning.

|  | **d9** | |  |  | **d21** | |  |  |
| --- | --- | --- | --- | --- | --- | --- | --- | --- |
| **Metabolite** | **BBW class^1^** | | **SEM** | **P-value^2^** | **BBW class^1^** | | **SEM** | **P-value^2^** |
|  | **LBBW** | **NBBW** |  |  | **LBBW** | **NBBW** |  |  |
| 2-Oxoglutarate | 0.38 | 0.29 | 0.04 | 0.34 | 0.31 | 0.27 | 0.02 | 0.52 |
| 3-Aminoisobutyrate | 0.15 | 0.17 | 0.01 | 0.22 | 0.15 | 0.12 | 0.02 | 0.63 |
| Acetate | 0.26 | 0.29 | 0.01 | 0.68 | 0.17 | 0.14 | 0.01 | 0.83 |
| Acetone | 0.02 | 0.02 | 0.0002 | 0.54 | 0.02 | 0.03 | 0.002 | 0.76 |
| Arabinose | 0.11 | 0.13 | 0.01 | 0.81 | 0.11 | 0.1 | 0.01 | 0.84 |
| Arginine | 0.17 | 0.15 | 0.01 | 0.47 | 0.18 | 0.21 | 0.02 | 0.18 |
| Ascorbate | 0.08 | 0.09 | 0.01 | 0.87 | 0.08 | 0.07 | 0.004 | 0.48 |
| Asparagine | 1.11 | 0.76 | 0.17 | 0.14 | 1.17 | 1.21 | 0.02 | 0.84 |
| Betaine | 0.72 | 0.69 | 0.01 | 0.70 | 1.41 | 1.58 | 0.08 | 0.32 |
| Citramalate | 0.11 | 0.13 | 0.01 | 0.13 | 0.13 | 0.16 | 0.01 | 0.79 |
| Citrate | 0.72 | 0.67 | 0.02 | 0.24 | 0.60 | 0.61 | 0.004 | 0.68 |
| Creatine | 0.82 | 1.02 | 0.10 | 0.47 | 1.00 | 0.49 | 0.26 | 0.14 |
| Fumarate | 0.01 | 0.01 | 0.0003 | 0.64 | 0.01 | 0.01 | 0.002 | 0.16 |
| Glucose | 22.7 | 22.4 | 0.15 | 0.76 | 16.8 | 19.2 | 1.2 | 0.24 |
| Glutamine | 2.97 | 3.31 | 0.17 | 0.18 | 2.31 | 2.64 | 0.16 | 0.20 |
| Glycerol | 0.65 | 0.66 | 0.004 | 0.88 | 0.91 | 0.84 | 0.03 | 0.75 |
| Isoleucine | 0.42 | 0.46 | 0.02 | 0.19 | 0.49 | 0.42 | 0.03 | 0.18 |
| Lysine | 0.08 | 0.08 | 0.002 | 0.84 | 0.06 | 0.06 | 0.0012 | 0.46 |
| Methanol | 0.05 | 0.07 | 0.01 | 0.15 | 0.07 | 0.17 | 0.05 | 0.75 |
| Methionine | 0.13 | 0.15 | 0.01 | 0.49 | 0.09 | 0.10 | 0.01 | 0.47 |
| myo-Inositol | 0.63 | 0.69 | 0.03 | 0.44 | 0.48 | 0.49 | 0.01 | 0.87 |
| N,N-Dimethylglycine | 0.03 | 0.02 | 0.004 | 0.38 | 0.04 | 0.04 | 0.0002 | 0.74 |
| Phenylalanine | 0.24 | 0.26 | 0.01 | 0.27 | 0.19 | 0.14 | 0.03 | 0.15 |
| Proline | 0.80 | 0.74 | 0.03 | 0.49 | 0.60 | 0.65 | 0.02 | 0.41 |
| Sarcosine | 0.11 | 0.08 | 0.01 | 0.17 | 0.14 | 0.15 | 0.002 | 0.94 |
| Succinate | 0.04 | 0.03 | 0.003 | 0.29 | 0.06 | 0.06 | 0.0004 | 0.72 |
| TMAO | 0.02 | 0.03 | 0.001 | 0.61 | 0.04 | 0.05 | 0.003 | 0.55 |

**^1^** the class of birth body weight was divided into 2 groups: normal birth body weight (NBBW: 16 piglets, 1.38±0.09kg) and low both body weight (LBBW: 16 piglets, 0.92±0.07kg). ^2^ Statistical analysis were performed as follow: linear mixed model and ANOVA analysis in which the class of BBW was included as a fixed factor, and litter of origin was included as a random factor. Piglets were used as experimental unit. Prior to analyses, the log, or Box and Cox transformation was performed to normalize the data distribution.


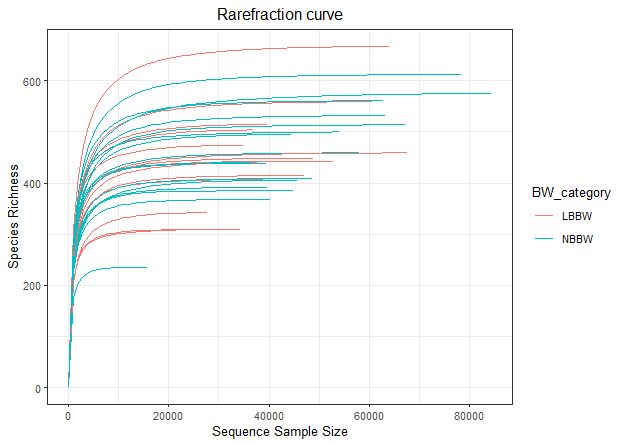


**Supplementary Figure S1.** Rarefaction curve of piglets’ caecal samples resulted by sequencing of 16S V3–V4 regions with MiSeq platform (Illumina Inc., San Diego, CA, USA). The class of birth body weight was divided into 2 groups: normal birth body weight (NBBW: 16 piglets, 1.38±0.09kg) and low both body weight (LBBW: 16 piglets, 0.92±0.07kg).


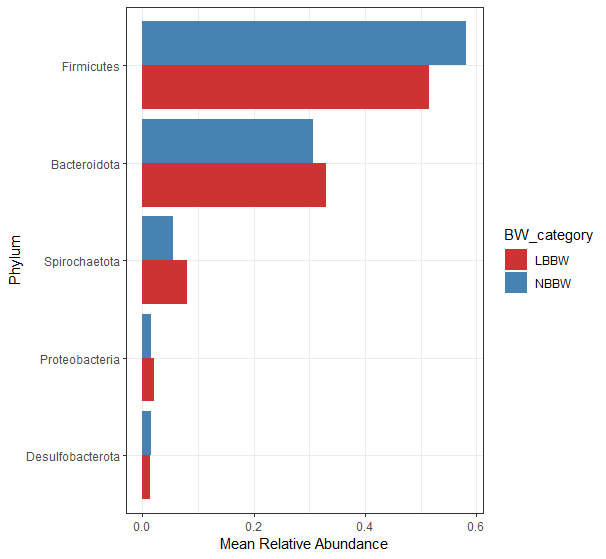

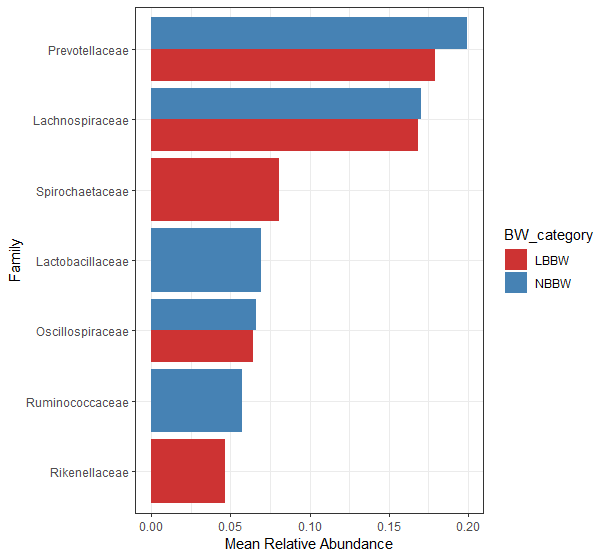

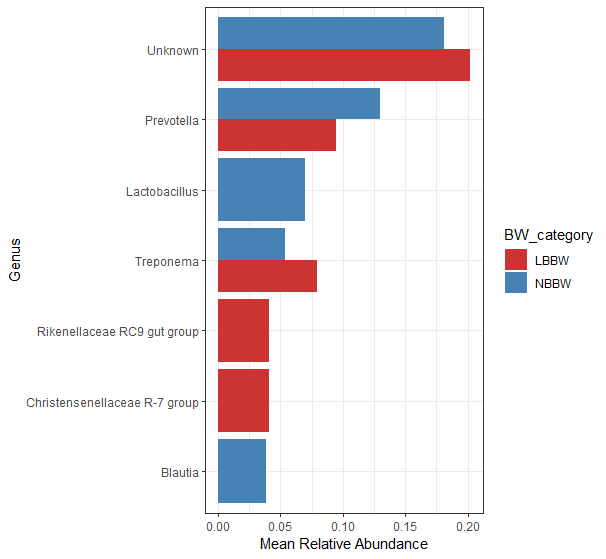


A

C

B

**Supplementary Figure S2.** Effect of birth body weight on microbial composition at Phylum (A), Family (B), and Genus level (C) of weaned piglets at two timepoints; the class of birth body weight was divided into 2 groups: normal birth body weight (NBBW: 16 piglets, 1.38±0.09kg) and low both body weight (LBBW: 16 piglets, 0.92±0.07kg).
